# Digital multicriteria evaluation of negotiated medicines using Chinese mini-HTA: application of structured methodologies in assessing once-weekly GLP-1 RAs for improved clinical decision-making

**DOI:** 10.3389/fphar.2025.1588056

**Published:** 2025-09-17

**Authors:** Xiao Li, Zhihong Qiu, Chaojun Xue, Xiaokai Ren, Zhanjun Dong

**Affiliations:** ^1^ Department of Pharmacy, Hebei General Hospital, Shijiazhuang, Hebei, China; ^2^ Hebei Key Laboratory of Clinical Pharmacy, Shijiazhuang, Hebei, China; ^3^ School of Clinical Pharmacy, Hebei Medical University, Shijiazhuang, Hebei, China

**Keywords:** selection evaluation, digital evaluation, national health insurance negotiated medicines, once-weekly GLP-1 RAs, mini HTA

## Abstract

**Background:**

Systematic and transparent evaluation of medicines remains a global challenge. In China, structured frameworks for clinical value assessment are underutilized despite improved access to negotiated medicines. Once-weekly glucagon-like peptide-1 receptor agonists (GLP-1 RAs) were selected as a representative case for their reimbursement status and clinical and economic relevance. This study applied a quantitative mini-health (mini-HTA) technology assessment to support rational drug selection.^1^

**Methods:**

A structured, three-stage methodology was employed to evaluate four once-weekly GLP-1 RAs. First, a weighted scoring system was established for five dimensions—pharmaceutical properties, effectiveness, safety, economy, and other considerations—based on expert consensus using the Quantitative Record Form for Drug Evaluation and Selection in Medical Institutions. Second, evidence for each dimension was systematically collected using a PICO-based search strategy across guideline databases, literature sources, and official documents. Third, each drug was quantitatively scored in each dimension according to predefined criteria and expert-assigned weights; the total scores were used to classify drugs into recommendation levels (“strongly recommended,” “weakly recommended,” or “not recommended”) to guide evidence-based selection in medical institutions.

**Results:**

Semaglutide (77.8) and dulaglutide (76.3) achieved the highest totals, driven by superior HbA1c reduction and proven cardiovascular benefit, and were strongly recommended. Exenatide microspheres scored 70.2, mainly owing to favourable acquisition cost, and was also strongly recommended. PEG loxenatide scored 62.9, limited by narrower reimbursement coverage and lower international uptake, and received a weak recommendation. Safety profiles were comparable across agents.

**Conclusion:**

The study demonstrates that a structured, expert-informed mini-HTA framework can be feasibly applied for quantitative evaluation and selection of once-weekly GLP-1 RAs in Chinese medical institutions. Key differentiators among agents were efficacy (notably cardiovascular benefit) and economic/policy factors, while safety differences were minimal. This replicable approach improves transparency, consistency, and evidence-based decision-making in clinical pharmacy and institutional formulary management.

## 1 Introduction

The global healthcare landscape continues to grapple with persistent challenges in the evaluation and selection of medicines. Delivering the appropriate medicine to the right patient requires assessing factors influencing individual drug responses. In this context, the Chinese government has undertaken significant efforts to improve the accessibility and affordability of negotiated medicines (innovative drugs that have been added to the National Reimbursement Drug List in China through formal price negotiations with the National Healthcare Security Administration, thereby improving their affordability and accessibility). A notable initiative is the issuance of the *Circular on Further Regulating the Use of Negotiated Drugs* by the National Health Security Administration and the National Health Commission. This directive urges medical institutions to stock and promptly utilize negotiated drugs to improve accessibility. Concurrently, the Department of Pharmaceutical Administration issued the *Circular of the General Office of the National Health Commission on Standardizing the Work of Comprehensive Clinical Evaluation of Medicines*, which underscores the importance of rigorous clinical evaluations. These efforts align with elevated public expectations for healthcare quality and global initiatives such as the *World Health Organization Model List of Essential Medicines*. For over 4 decades, the *World Health Organization Model List of Essential Medicines* has served as a critical guide for country-level medicine selection and financing, supporting universal health coverage and access to essential medicines worldwide. Health technology assessment (HTA) has become an increasingly prominent tool for healthcare agencies globally, informing decision-making regarding the adoption of new health technologies, including drug selection. Among the various HTA approaches, the mini-HTA has emerged as a particularly effective hospital-based tool. This method enables rapid assessment across four critical dimensions: technology, patient, organization, and economy. By addressing these dimensions, mini-HTA supports both political and clinical decision-making at all levels of the healthcare system.

**FIGURE 1:**
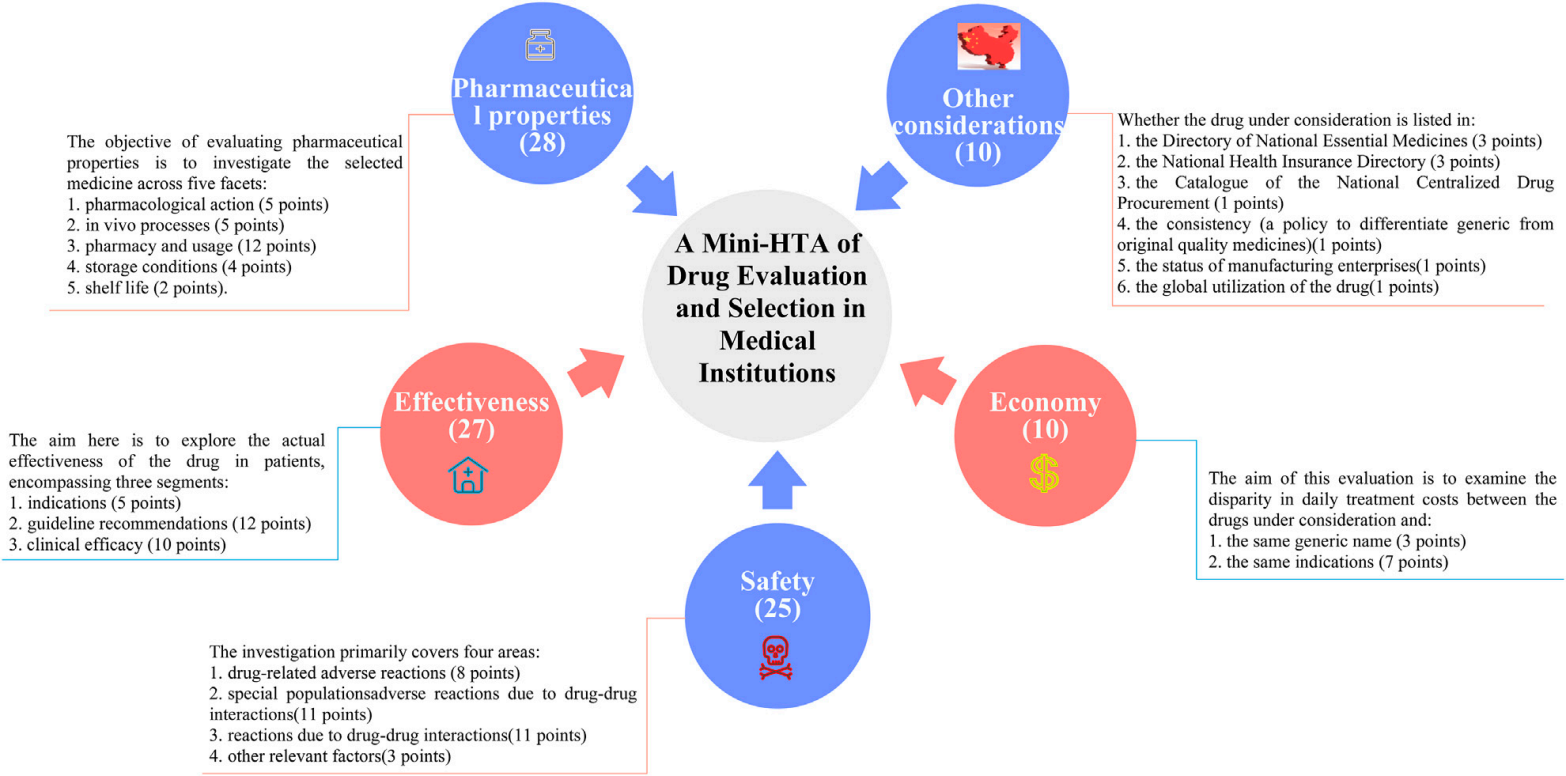
The overall drug evaluation system.

In China, the growing focus on drug selection and evaluation in certain provinces and cities has underscored the urgent need for medical institutions to develop comprehensive and quantifiable drug selection and evaluation systems. In April 2023, a revised guideline titled *A Quick Guideline for Drug Evaluation and Selection in Chinese Medical Institutions (Second Edition)* was introduced as a hospital-based mini-HTA tool. This guideline incorporates the *Quantitative Record Form for Drug Evaluation and Selection in Medical Institutions* (Figure 1), which aligns closely with national policy mandates (Martelli et al., 2016). The quantitative record form defines five distinct dimensions for pharmaceutical assessment: pharmaceutical properties, effectiveness, safety, economy, and other considerations. Each dimension is quantified and scored, enabling an objective evaluation and selection of drugs within medical institutions. By translating drug attributes into numerical values, the evaluation scores provide hospital decision-makers with actionable insights to guide drug selection processes (Wong-Rieger et al., 2025). While this structured approach significantly influences resource allocation decisions, its application in clinical settings remains a topic of ongoing debate. The use of a digital selection and rating table facilitates a detailed evaluation of negotiated medicines across multiple dimensions and levels of evidence. This approach not only enhances the supply assurance of negotiated medicines but also promotes their reintegration into clinical practice. Such advancements are particularly critical given the high prevalence of diabetes in China.

China currently has the highest number of individuals with diabetes globally (Zhoa et al., 2023). Glucagon-like peptide-1 receptor agonists (GLP-1 RAs) play a pivotal role in diabetes management by stimulating the production of glucagon-like peptide-1. This peptide binds to specific receptors on pancreatic β-cells, promoting insulin secretion and thereby reducing blood glucose levels. GLP-1 RAs are associated with a lower risk of hypoglycemia and offer cardiovascular benefits, leading to improved clinical outcomes (Author anonymous, 2021). Among these, once-weekly GLP-1 RAs have gained widespread recognition for their notable safety profile, efficacy, and convenience (Linong et al., 2018). Currently, four types of once-weekly GLP-1 RAs are available in the Chinese market: exenatide microspheres, dulaglutide, polyethylene glycol (PEG) loxenatide, and semaglutide. Notably, the semaglutide injection, introduced in 2022, has emerged as a promising hypoglycemic agent with established cardiovascular benefits (American Diabetes Association, 2022). We therefore selected once-weekly GLP-1 RAs as the case for evaluating the Chinese mini-HTA methodology, as they represent a clinically important, policy-relevant, and multidimensional class of medicines that can comprehensively test the framework’s applicability.

Despite these advancements, there remains a significant lack of comprehensive studies utilizing the well-structured quantitative record form to appraise the clinical value of once-weekly GLP-1 RAs. This gap in evidence limits the ability of medical institutions to make fully informed decisions regarding drug selection. This study addresses this gap by systematically evaluating the clinical value of once-weekly GLP-1 RAs using the quantitative record form. By detailing the implementation process, this study seeks to contribute to the broader goals of improving drug selection, optimizing resource allocation, and enhancing patient care in diabetes management within China. Furthermore, it aspires to offer insights and methodologies that could be adapted globally to improve drug evaluation and selection processes. The results of the study have implications for addressing global healthcare challenges. Specifically, the implementation of small healthcare assessment frameworks and quantitative record sheets may serve as effective models for other countries, particularly those experiencing similar healthcare constraints. Furthermore, the adoption of structured drug evaluation approaches can help to address issues related to the affordability and accessibility of medicines in low- and middle-income countries.

## 2 Methods

This study employs a structured, three-stage methodology comprising the evaluation system, evidence collection, and comprehensive analysis and decision-making stages. The initial stage uses a well-defined evaluation system to categorize and assess the drugs under investigation. In the second stage, a rigorous evidence collection process is conducted, during which relevant literature and data are gathered and analyzed to inform the evaluation. In the final stage, evidence is interpreted, scores are assigned to the drugs, and recommendations are made regarding their inclusion in medical institutions.

### 2.1 Evaluation system stage

**FIGURE 2:**
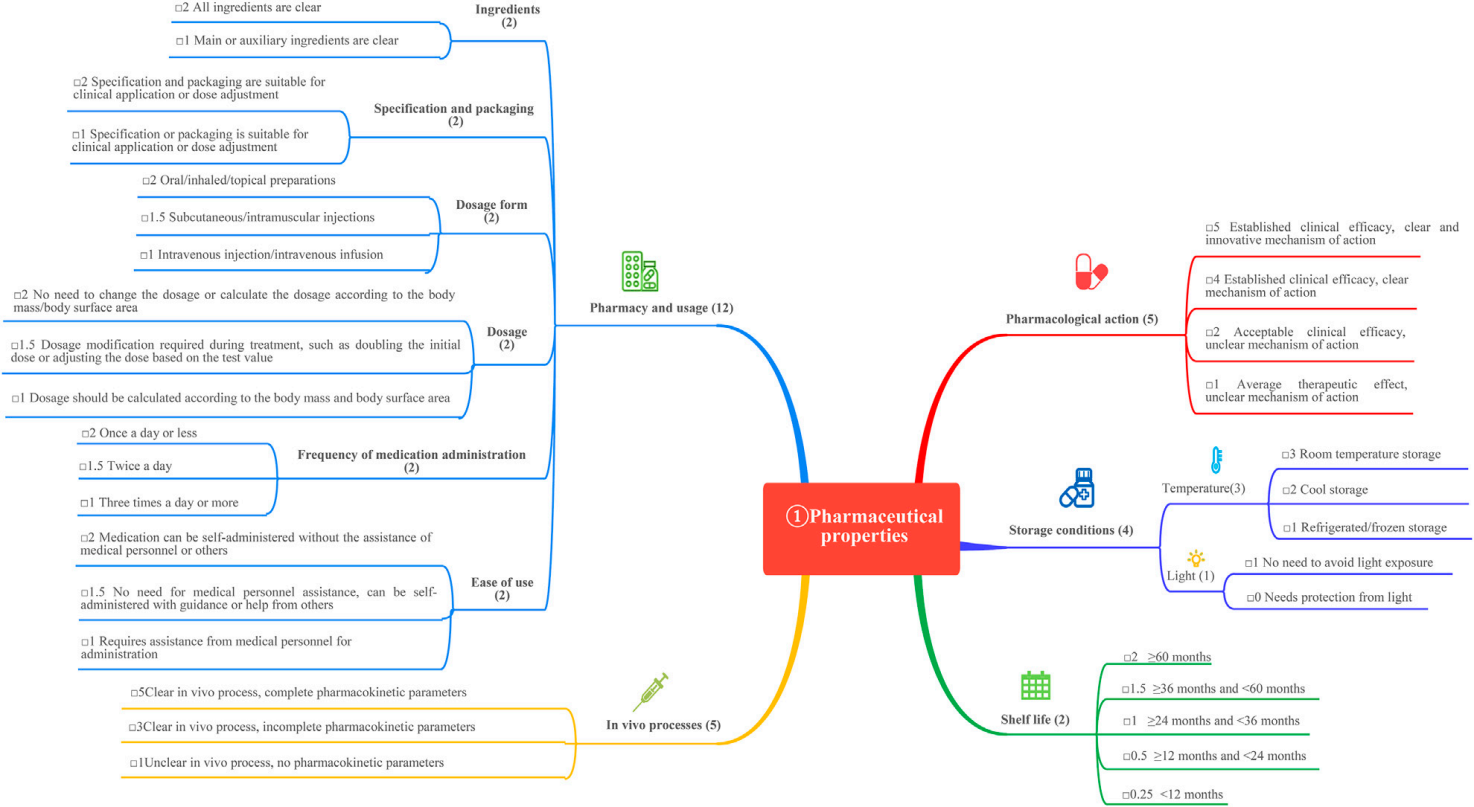
The “pharmaceutical properties” evaluation.

**FIGURE 3:**
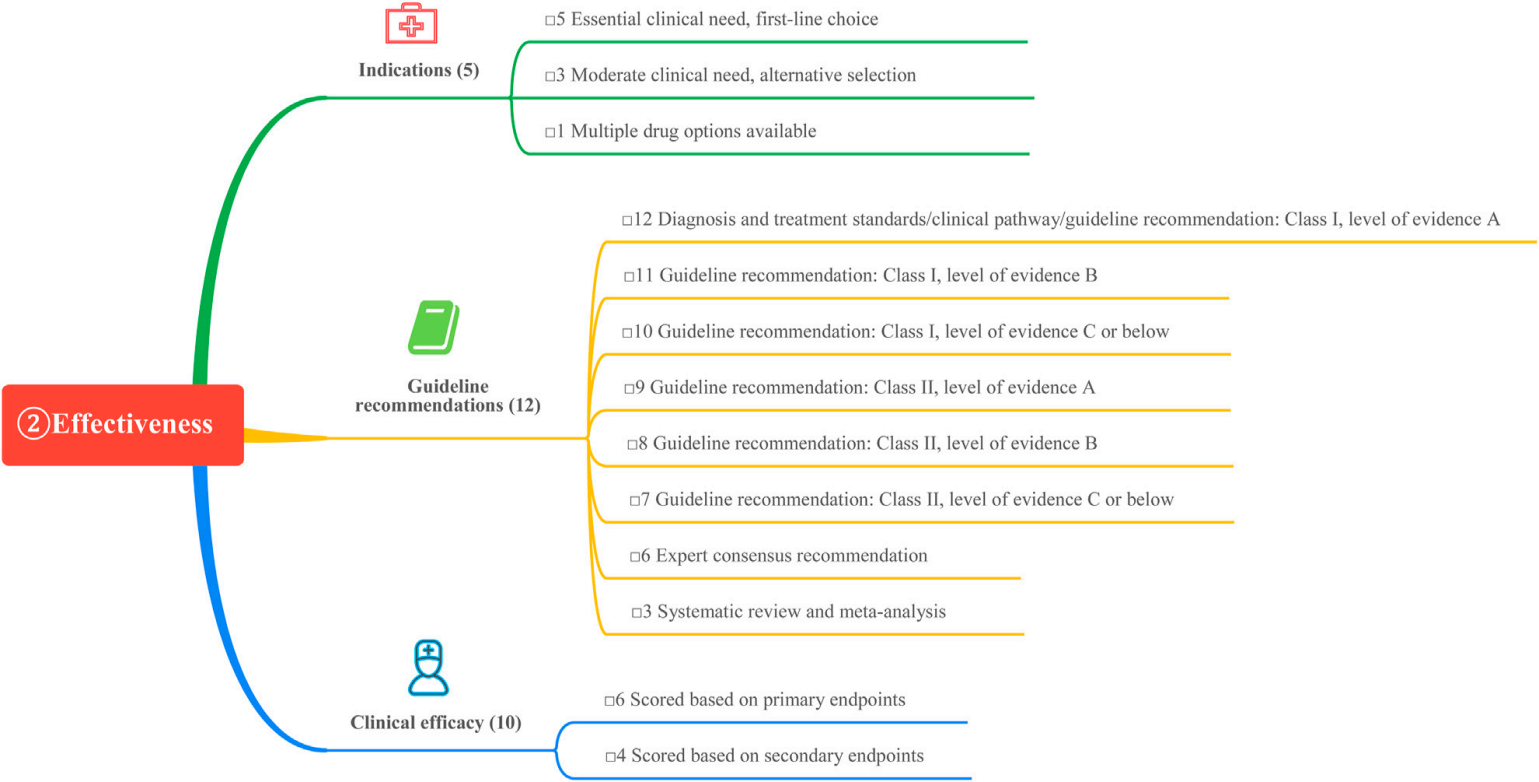
The “effectiveness” evaluation system.

**FIGURE 4:**
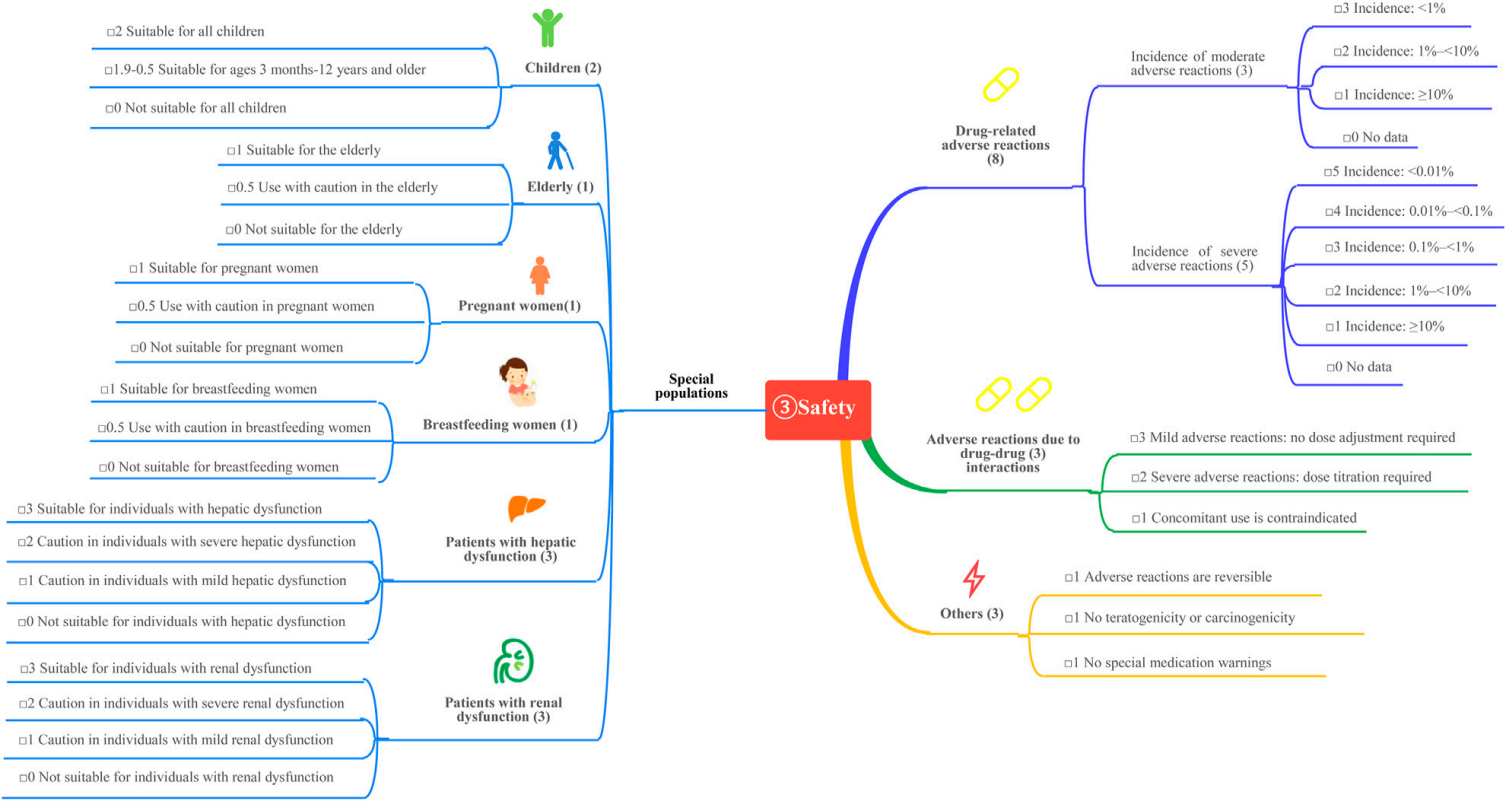
The “safety” evaluation system.

**FIGURE 5:**
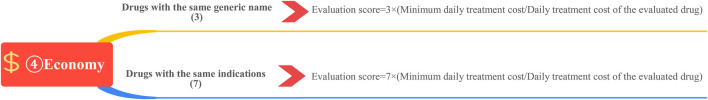
The “economy” evaluation system.

**FIGURE 6:**
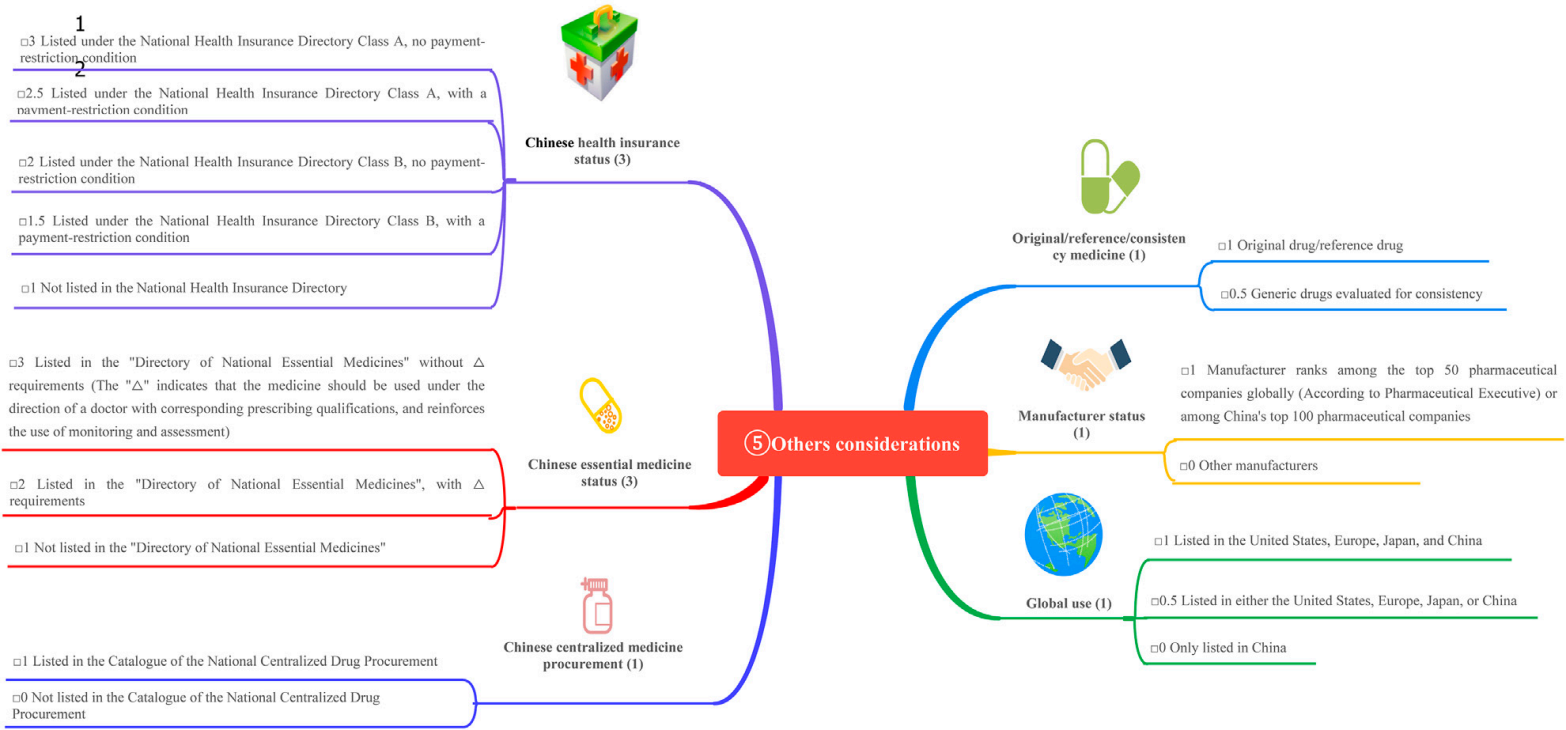
The “others considerations” evaluation system.

This study is based on the *Quantitative Record Form for Drug Evaluation and Selection in Medical Institutions* (Figure 1), outlined in *A Quick Guideline for Drug Evaluation and Selection in Chinese Medical Institutions* (*Second Edition*; hereafter referred to as *the Guideline*). This form is based on evidence-based research findings (Martelli et al., 2016; Mannucci et al., 2020) and integrates the Mini-HTA assessment framework with the System of Objectified Judgement Analysis method, which has been adapted to align with China’s unique national context (Marsico et al., 2020). The evaluation system is structured around five core dimensions: pharmaceutical properties, effectiveness, safety, economy, and other considerations. Each dimension is assigned a specific weight to reflect its relative importance in the overall assessment: pharmaceutical properties (28 points, Figure 2), effectiveness (27 points, Figure 3), safety (25 points, Figure 4), economy (10 points, Figure 5), and other considerations (10 points, Figure 6). The total score for the evaluation indices is 100 points, ensuring a comprehensive and balanced assessment framework.

The assignment of weights for each evaluation dimension was accomplished using the Delphi method through anonymous online voting via the Wenjuanxing online platform. A total of 76 participants, including members from the steering (n = 18) and expert panels (n = 58) with multidisciplinary backgrounds in pharmacy, clinical medicine, health policy, and pharmacoeconomics, contributed to the process. Additionally, an external review group of 20 experts (comprising pharmacists, representatives from medical insurance offices, social security bureaus, academic associations, and schools of pharmacy) provided Supplementary Input and independent assessment.

During the Delphi process, participants anonymously rated and assigned weights to each evaluation indicator. Two rounds of online discussions—including both the expert panel and the external review group—were held to discuss and refine the rationality and appropriateness of the indicators and their assigned weights. Consensus was defined as at least 75% agreement among participants for each item, in line with commonly accepted standards in Delphi methodology (Barrios et al., 2021). All experts declared no relevant conflicts of interest before participation. This approach ensured that the final weighting system accurately reflected both evidence and the specific priorities within China’s healthcare context, as shown in Figures 1–6.

Final weights were calculated using a normalized scoring approach to ensure the sum of all weights equaled 100 points, according to the following formula:

$$W=\frac{\sum_{i=1}^{k}W_{i}}{k}$$

where *k* is the number of participants in the index weight evaluation, *k* = 76.

The weights for each dimension were calculated using a normalized scoring approach. Specifically, the sum of the individual weightings was normalized to ensure the total score sums to 100 points. The formula for calculating index weight is as follows:

$$\text{Weight}=\left(\text{Raw Weight of Dimension }/\text{ Sum of All Weights}\right)\times 100$$

The weights of the five evaluation indicators were illustrated in Table 1.

**TABLE 1:** The weights of the five evaluation indicators.

Index weight	Pharmaceutical properties	Effectiveness	Safety	Economy	Other considerations
*W*	28	27	25	10	10

### 2.2 Evidence collection stage

#### 2.2.1 Search strategy

The PICO framework (participants, interventions, comparisons, and outcomes) was utilized to define research topics and keywords, enabling the development of a systematic and comprehensive search strategy. Selected keywords included “semaglutide”, “dulaglutide”, “exenatide microspheres”, “PEG loxenatide”, “GLP-1 RA”, and “glucagon-like peptide-1 receptor agonist”.

#### 2.2.2 Scope of search

(1) Guideline databases: US National Clinical Guidelines Database, International Guidelines Collaboration Network, Trip Guideline Database, and the UK National Institute for Health and Clinical Excellence. (2) English and Chinese literature databases: PubMed, The Cochrane Library, Embase, Wanfang Database, VIP Database, Chinese Biomedical Literature Database, and Chinese Journal Full Text Database. (3) Official websites: Portals of international and national health administrations, drug regulatory authorities, and relevant industry associations. (4) Drug instructions: Official documentation provided by pharmaceutical manufacturers.

#### 2.2.3 Inclusion and exclusion criteria

The inclusion and exclusion criteria were established to ensure the selection of high-quality and relevant literature. Regarding pharmaceutical properties, studies addressing pharmacological action, *in vivo* processes, pharmacy and usage, storage conditions, and shelf life were included, while meeting abstracts and duplicate studies were excluded. For effectiveness and safety, the inclusion criteria focused on multi-center randomized controlled trials conducted internationally or single-center studies in China, as well as guidelines endorsing the use of four once-weekly GLP-1 receptor agonists (GLP-1 RAs). Conversely, trials involving substances other than the four once-weekly GLP-1 RAs and guidelines that did not recommend their use were excluded. Multi-center RCTs were prioritized for inclusion due to their higher internal validity and ability to minimize bias. Single-center studies from China were included to capture local data, though these studies were assessed for potential bias using a standardized tool.

#### 2.2.4 Other data sources

**FIGURE 7:**
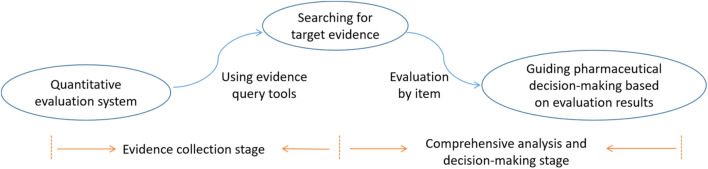
Selection and evaluation implementation roadmap.

To supplement the search, additional data were gathered from various sources. Drug prices were obtained from the Hebei Provincial Medical Institutions Drug Trading and Purchasing Platform, while information on medical insurance attributes was referenced from the National Drug Catalog for Basic Medical Insurance, Work Injury Insurance, and Maternity Insurance (2024 edition). Data on essential drug properties were derived from the National Essential Drugs Catalog (2018 edition). Global utilization data were collected from PharmaSmart.com and the websites of drug administration departments in various countries. Corporate credibility assessments were based on information from the Pharm Exec (2024 edition), as shown in Figure 7. It is important to note that all data sources used in this study were secondary in nature, including published clinical trials, clinical guidelines, official documents, and policy databases. No individual-level primary real-world data (RWD)—such as electronic health records, insurance claims, or patient registries—were incorporated. Instead, all data were extracted from publicly available, structured sources to ensure reproducibility and transparency.

### 2.3 Comprehensive analysis and decision-making stage

The evidence collected during the previous stage was systematically organized in alignment with the indicators defined in the evaluation system. Each of the four once-weekly GLP-1 receptor agonists (GLP-1 RAs) was assigned a score for each indicator based on evidence-based data. To ensure fairness and scientific rigor, the scoring process was conducted in multiple rounds. In the first round, two clinical pharmacists with relevant expertise independently evaluated each drug according to the five established dimensions and assigned initial scores. In the second round, a multidisciplinary expert panel—including the director of the pharmacy department, 35 pharmacists, 3 physicians, and 3 nurses—reviewed and discussed the initial scores and the supporting evidence. Based on the panel’s discussion, the scores were revised to reflect group consensus. Finally, a further discussion was held to confirm and finalize the scores for each drug. These individual scores were then aggregated to calculate total scores for the four GLP-1 RAs, which served as the basis for pharmaceutical decision-making.

Recommendations for new drug inclusion were divided into three groups based on total scores. Specifically, the categorization thresholds were directly adopted from *the Guideline* (Zhao et al., 2023): a total score of ≥70 was defined as a strong recommendation for inclusion in the institutional drug list by the Committee of Drug Administration and Pharmacotherapeutics; a score between 60 and 69 corresponded to a weak recommendation or non-recommendation depending on the availability of therapeutic alternatives; and a score <60 indicated a non-recommendation for inclusion. This systematic approach ensures a rigorous, evidence-based evaluation and recommendation process for new drugs. By addressing the critical need for informed pharmaceutical decision-making within medical institutions, it enhances both the quality and efficiency of the drug selection process. Individual scores for each dimension (pharmaceutical properties, effectiveness, safety, economy, and other considerations) were aggregated using the following weighted average formula:

$$\text{Total Score}=\left(\text{Score for Pharmaceutical Properties}\times \text{Weight for Pharmaceutical Properties}\right)+\left(\text{Score for Effectiveness}\times \text{Weight for Effectiveness}\right)+\ldots$$

This aggregation method ensured that the final score appropriately reflected the relative importance of each dimension.

## 3 Results

### 3.1 Pharmacological properties of GLP-1RAs action

**FIGURE 8:**
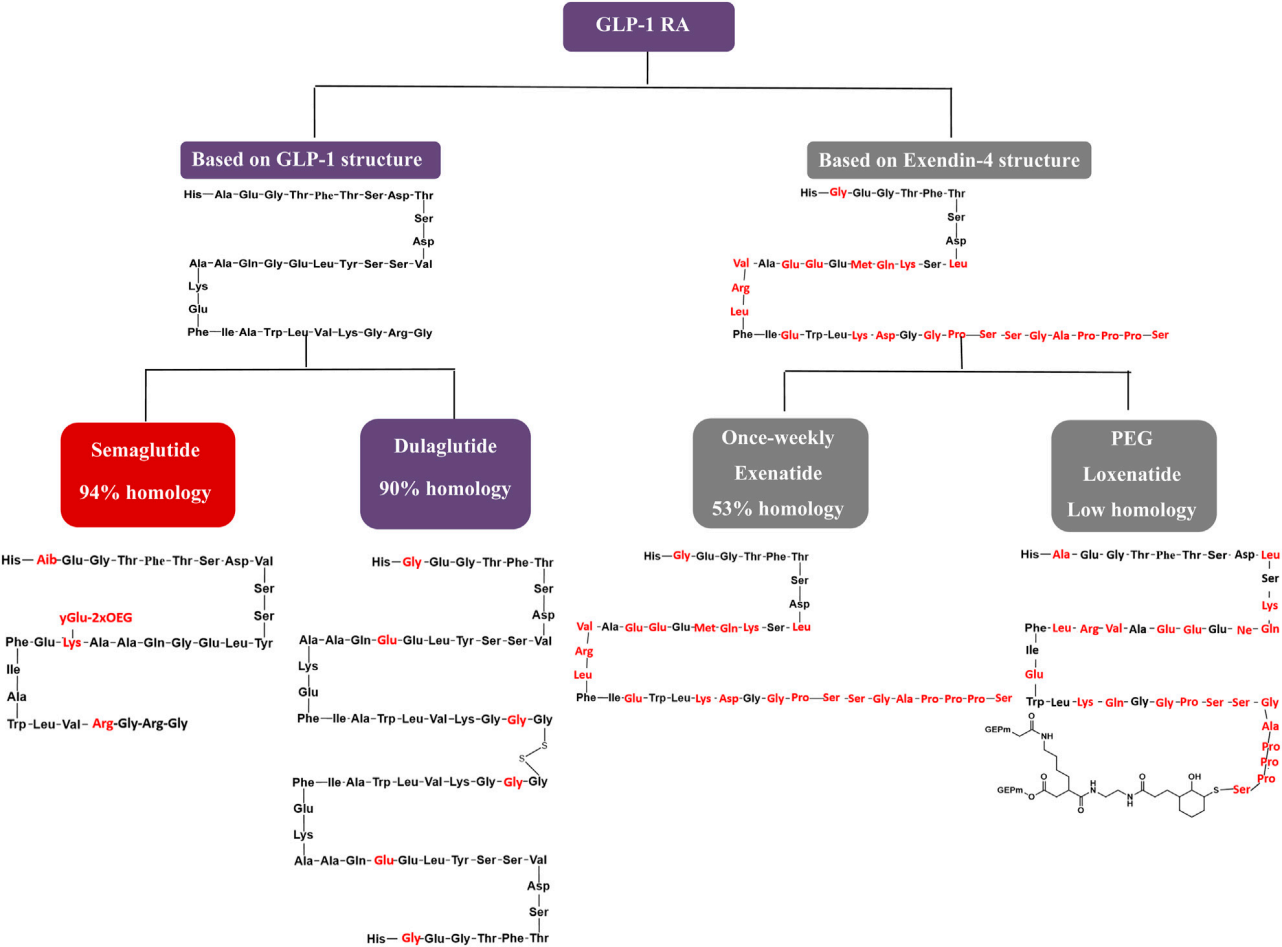
Comparison of the molecular structure of once-weekly GLP-1 RAs.

The GLP-1 RAs exert antihyperglycemic effects by enhancing glucose-dependent insulin secretion (Zhao et al., 2020). Weekly GLP-1 RAs formulations are categorized into two groups based on their structural modifications. The first group involves localized modifications to the molecular structure of GLP-1, resulting in high homology with native GLP-1 and a low incidence of allergic reactions during clinical use (Manandhar and Ahn, 2015). The second group comprises structural modifications of exenatide (exendin-4), a GLP-1 analogue with lower homology to GLP-1, which is associated with a higher incidence of allergic reactions in clinical practice. Focusing on structural modifications, semaglutide achieves 94% homology with native GLP-1 by substituting three amino acids at positions 8, 26, and 34. This homology surpasses dulaglutide and significantly exceeds exenatide-based formulations and PEG loxenatide (Lund et al., 2014; Lau et al., 2015). This structural similarity to native GLP-1, particularly in semaglutide and dulaglutide, contributes to enhanced receptor binding affinity and prolonged resistance to enzymatic degradation, supporting longer half-life and once-weekly dosing. In contrast, exenatide and PEG loxenatide, with lower homology, display different pharmacokinetic profiles that may influence efficacy and clinical use. Figure 8 highlights these molecular differences, linking structure to pharmacological behavior and clinical performance.

As depicted in the molecular structure formula, semaglutide has the smallest molecular formula among weekly GLP-1 RA preparations, with a molecular weight of 4.1 kDa. This smaller molecular size facilitates *in vivo* absorption, enhancing its hypoglycemic efficacy. Semaglutide extends its action time to 7 days by incorporating fatty acid side chains, unlike the microglobulin technology in exenatide, the macromolecular linker protein in dulaglutide, or the pegylated linker in PEG loxenatide (Lund et al., 2014). Due to its well-documented clinical efficacy and clear mechanism of action (Zhao et al., 2020), semaglutide stands out with the lowest molecular weight and highest homology in its class, earning a score of 5, while the other agents receive a score of 4.

#### 3.1.1 *In vivo* processes

Studies have shown a direct correlation between half-life duration and fluctuations in drug concentration during the same dosing interval (Kapitza et al., 2015). With a 7-day half-life, semaglutide outperforms other weekly GLP-1 RAs in maintaining stable drug concentrations (Pradhan et al., 2020). Its pharmacokinetic parameters are precise and well-characterized, with a superior elimination half-life and stability, warranting a score of 5. In comparison, dulaglutide, exenatide microspheres, and PEG loxenatide exhibit clear but incomplete pharmacokinetic data, earning a score of 3 each, as shown in Table 2.

**TABLE 2:** Summary of pharmacokinetic parameters of four GLP-1 RAs.

Pharmacokinetic parameters		Semaglutide	Dulaglutide	Exenatide microspheres	PEG loxenatide
Absorption	T_max_ (h)	24–72	48	336 (hydration) 1,176 (corrosion)	67
	T_ss_ (w)	4–5	2–4	6–7	—
	C_max_ (g/L)	65.8 (0.5 mg qw) 123.4 (1 mg qw)	0.114 (1.5 mg qw)	300 (2 mg qw)	
	F (%)	89	47–65		70
Distribution	Vd (L)	12.5	19.2–17.4	28.3	2.72–5.43
	PPBR	>99%	—	—	—
Metabolism	T_1/2_ (h)	168	108–112.8		104–121
	DMEs	NEP	Protein catabolism		
Excretion	Cl/F (L/h)	0.05	0.107–0.111	9.1	0.015–0.02
	Pathway	2/3 Urine 1/3Faeces	—	Renal	Renal
Score		5	3	3	3

#### 3.1.2 Pharmacy and usage

The pharmacy and usage characteristics of the four GLP-1 RAs were evaluated using six indicators:

1. Ingredients: The ingredients of semaglutide, dulaglutide, exenatide microspheres, and PEG loxenatide are well-defined, earning each drug a score of 2.
2. Specification and packaging: Semaglutide, dulaglutide, and PEG loxenatide are available in both larger and smaller specifications, while exenatide microspheres are limited to a fixed specification. All drugs meet packaging requirements, with each receiving a score of 2.
3. Dosage form: All four drugs are administered via subcutaneous injection, earning a score of 1.5.
4. Dosage: Semaglutide, dulaglutide, and PEG loxenatide allow for dosage adjustments based on blood glucose levels, meriting a score of 1.5 each. Exenatide microspheres, with its fixed dosage, receives a score of 2.
5. Frequency of medication administration: All drugs are recommended for once-weekly subcutaneous injection at any time of day, earning a score of 2 each.
6. Ease of use: After physician instruction, all four GLP-1 RAs can be self-administered at home, demonstrating ease of use. Each drug is allocated a score of 1.5.

The pharmacy and usage indicators for semaglutide, dulaglutide, exenatide microspheres, and PEG loxenatide are summarized in Table 3, detailing their ingredients, specifications, dosage forms, and other relevant characteristics.

**TABLE 3:** Summary of pharmacy and usage of four GLP-1 RAs.

Pharmacy and usage indicators		Semaglutide	Dulaglutide	Exenatide microspheres	PEG loxenatide
Ingredients	Major	Semaglutide	Dulaglutide	Exenatide	PEG loxenatide
	Excipients	Na_2_HPO_4_·2H_2_O C_3_H_8_O_2,_ propofol HCl NaOH	C_6_H_6_Na_2_O_7_ C_6_H_8_O_7_ C_6_H_14_O_6_ Polysorbate 80 WFI	PLGA CMC NaCl Polysorbate 20, NaH_2_PO_4_·H_2_O, Na_2_HPO_4_·7H_2_O, WFI	CH_3_COONa, CH_3_COOH C_6_H_14_O_6_ WFI
Score		2	2	2	2
Specification and packaging		1 mL: 1.34 mg,1.5 mL 1 mL: 1.34 mg,3 mL	500 μL: 750 μg 500 μL: 1.5 mg	2 mg	0.5 mL:0.1 mg 0.5 mL:0.2 mg
Score		2	2	2	2
Dosage form		subcutaneous injection			
Score		1.5			
Dosage		Initial dosage: 0.25 mg 4 weeks later: 0.5 mg Another 4 weeks:1.0 mg	Initial dosage: 0.75 mg Max dosage:1.5 mg	2 mg	Initial dosage:0.1 mg Max dosage:0.2 mg
Score		1.5	1.5	2	1.5
Frequency of medication administration		Once a week			
Score		2			
Ease of use		Post-physician instruction, semaglutide can be self-administered at home, reflecting a relatively straightforward usage procedure			
Score		1.5			
Total score		10.5	10.5	11	10.5

#### 3.1.3 Storage conditions and shelf life

All four drugs require cold storage and protection from light, earning a score of 1 each. Semaglutide and exenatide microspheres have a shelf life of 36 months, meriting a score of 1.5, while dulaglutide and PEG loxenatide have a shelf life of 24 months, earning a score of 1.

The final scores for pharmaceutical characteristics are as follows: semaglutide (23), dulaglutide (19.5), exenatide microspheres (20.5), and PEG loxenatide (19.5). Semaglutide’s superior pharmacological and pharmacokinetic properties earn it the highest score in this category.

### 3.2 Effectiveness of GLP-1RAs

#### 3.2.1 Indications

All four weekly GLP-1 RAs preparations can be used in combination with metformin or sulfonylureas to achieve glycemic control in patients with type 2 diabetes mellitus (T2DM). Loxenatide is approved in China as a monotherapy for glycemic control in T2DM patients. Notably, dulaglutide and semaglutide have received approval for reducing the risk of major cardiovascular adverse events in adults with T2DM and cardiovascular disease, underscoring their cardiovascular benefits. The 2024 American Diabetes Association (ADA) Diabetes Standards recommend that adults with T2DM who are at high risk for or already have atherosclerotic cardiovascular disease (ASCVD), heart failure, and/or chronic kidney disease should receive treatment plans incorporating sodium-glucose cotransporter-2 inhibitors (SGLT-2is) and/or GLP-1 RAs. These agents provide glycemic control while reducing cardiovascular and renal risks. GLP-1 RAs with proven cardiovascular and renal benefits, such as semaglutide and dulaglutide, are prioritized for these indications, each earning a score of 5. Other GLP-1 RAs, which serve as secondary options for clinical needs, are assigned a score of 3.

#### 3.2.2 Guideline recommendations

As the prevalence of diabetes continues to rise, treatment guidelines are consistently updated to reflect emerging evidence. GLP-1 RAs, as innovative hypoglycemic agents, increasingly feature in diabetes management guidelines. The 2024 ADA guidelines recommend GLP-1 RAs as the preferred first-line injectable agents for patients with T2DM and ASCVD or high cardiovascular risk factors, replacing insulin as the primary injectable glucose-lowering therapy. Cardiovascular complications remain the leading cause of disability and mortality in patients with T2DM. Since 2008, the U.S. Food and Drug Administration has mandated cardiovascular safety evaluations for new hypoglycemic agents prior to approval. Cardiovascular outcomes trials have demonstrated that GLP-1 RAs, including liraglutide, dulaglutide, and semaglutide, significantly reduce the risk of major adverse cardiovascular events (Marso et al., 2016a; Gerstein et al., 2019; Marso et al., 2016b). Meta-analyses confirm that GLP-1 RAs reduce adverse cardiovascular events, cardiovascular mortality, and all-cause mortality (Bethel et al., 2018).

The 2024 ADA guidelines recommend GLP-1 RAs and SGLT-2is, with or without metformin depending on glycemic control needs, as initial therapeutic agents for patients with T2DM and comorbid ASCVD, heart failure, or chronic kidney disease (American Diabetes Association, 2024). Similarly, the *Chinese Guidelines for the Prevention and Control of Type 2 Diabetes Mellitus* (*2020*) advocate for GLP-1 RAs or SGLT-2is in T2DM patients with ASCVD or cardiovascular risk factors, regardless of glycosylated hemoglobin (HbA1c) levels, provided no contraindications exist (Soci and ety, 2021). Additionally, the *Chinese Expert Consensus on the Diagnosis and Treatment of Cardiovascular Disease in Patients with Diabetes Mellitus* (*2021*) recommends metformin as the initial treatment for T2DM patients with cardiovascular disease, unless contraindicated. If metformin is contraindicated or not tolerated, GLP-1 RAs or SGLT-2is with established cardiovascular and renal benefits are advised as first-line agents (Yihong et al., 2021). Given their cardiovascular benefits, semaglutide and dulaglutide are the preferred agents in treatment guidelines, each receiving a score of 12. In contrast, exenatide and loxenatide, which lack cardiovascular benefits, are assigned a score of 9.

#### 3.2.3 Clinical efficacy

Semaglutide (1.0 mg weekly) demonstrated a reduction in HbA1c of 1.6% in clinical trials, earning the highest score of 10. The hypoglycemic efficacy of the other agents is summarized in Table 4.

**TABLE 4:** Comparison of hypoglycemic efficacy of GLP-1 RA monotherapy.

		Semaglutide	Dulaglutide	Exenatide microspheres	PEG loxenatide
Clinical trials		Yihong et al. (2021)	Chinese Diabetes and Chinese Diabetes Society (2020)	Ping and Jing (2022)	Sha et al. (2022)
Study period (weeks)		30	26	20	24
Sample size (persons)		388	807	121	361
Mean baseline level (HbA1c)/%		7.0–10.0	6.5–9.5	8.5	7–10.5
Comparison of glucose-lowering efficacy (HbA1c reduction)/%	Intervention group	1.6	0.78	1.54	1.34
	Control group	Placebo: 0.02	Metformin: 0.56	—	Placebo: 0.17
Score^a^		10	9.3	9.6	8.4

^a^ Scores are based on the reduction in HbA1c observed in clinical trials, with the agent achieving the largest reduction receiving the maximum score of 10.

In the SUSTAIN-7 clinical trial, semaglutide (1.0 mg weekly) demonstrated superior hypoglycemic efficacy over dulaglutide, earning the highest score of 10. Dulaglutide received a score of 9.3, exenatide 9.6, and loxenatide 8.4.

The total effectiveness scores for the four GLP-1 RAs are semaglutide (27), dulaglutide (26.3), exenatide (21.6), and loxenatide (20.4). Semaglutide demonstrated the highest overall effectiveness, driven by its superior indications, guideline recommendations, and clinical efficacy.

### 3.3 Adverse reactions and safety profile

#### 3.3.1 Drug-related adverse reactions

Clinical studies report no significant differences in adverse reaction incidence among GLP-1 RAs. The most reported adverse reactions are gastrointestinal symptoms, such as nausea and vomiting, which occur in over 10% of patients but are generally tolerable with prolonged treatment. When used as monotherapy, GLP-1RAs present a low risk of hypoglycemia. Moderate adverse reactions are assigned a score of 1. Serious adverse events, including pancreatitis, renal injury, cholecystitis, and venous thrombosis, have been reported in the literature; however, their incidence is less than 0.01% (Chinese Diabetes and Chinese Diabetes Society, 2020; Ping and Jing, 2022; Sha et al., 2022). These serious adverse events are assigned a score of 5. The total score for the seven GLP-1 RAs adverse events is 6.

#### 3.3.2 Special populations

Children: GLP-1 RAs are not approved for use in children or adolescents under 18 years of age with type 2 diabetes mellitus (T2DM) in China, resulting in a score of 0.

Elderly: Phase III clinical trials and meta-analyses have confirmed the efficacy and safety of GLP-1 RAs in individuals aged ≥65 years (Bode et al., 2011; Boustani et al., 2016), earning a score of 1.

Pregnant and Lactating Women: Exenatide may be considered during pregnancy if the potential benefits to the fetus outweigh the risks, earning a score of 0.3. The use of GLP-1 RAs in lactating women is recommended with caution, earning a score of 0.5. Other GLP-1 RAs lack clinical studies in pregnant women, have an unknown safety profile, and are not recommended. These agents are assigned a score of 0. Animal studies have shown that some GLP-1 RAs are secreted in breast milk, and thus, their use in lactating women is not recommended.

Patients with abnormal liver and kidney function: In patients with hepatic and renal dysfunction, dose adjustments and corresponding scores for GLP-1 RAs are summarized in Table 5.

**TABLE 5:** Dose adjustment and scoring in patients with hepatic and renal dysfunction for GLP-1 RA.

		Semaglutide	Dulaglutide	Exenatide microspheres	PEG loxenatide
Hepatic dysfunction	Mild to moderate	✓	✓	Unkown	Unkown
	Severe	✓	✓	Unkown	Unkown
	Score	3	3	0	0
Renal dysfunction	Mild	✓	✓	✓	✓
	Moderate	✓	✓	✓	Dose reduction
	Severe	✓ /Prohibited in end-stage renal disease	✓ /Prohibited in end-stage renal disease	×	×
	Score	2.9	2.9	2	1.5

Data sourced from the *Clinical Expert Consensus on Glucagon-like Peptide-1, Receptor Agonists for the Treatment of Type 2 Diabetes* (*2020 edition*) (Marso et al., 2016b) and drug labels. “√” indicates recommended use, while “ × ” indicates not recommended.

#### 3.3.3 Adverse reactions due to drug-drug interactions

GLP-1 RAs exhibit minimal drug-drug interactions. While the risk of hypoglycemia is low when used alone, the risk increases when combined with sulfonylureas or insulin. Additionally, GLP-1 RAs may interfere with the absorption of oral medications due to delayed gastric emptying. When combined with warfarin, more frequent monitoring of the international normalized ratio is recommended, earning a score of 3.

#### 3.3.4 Other adverse effects

GLP-1 RAs carry a risk of pancreatitis and thyroid cancer. The reversibility score for adverse effects is 0.8, while the carcinogenicity and teratogenicity scores are 0.

The safety scores for GLP-1 RAs are as follows: semaglutide (16.7), dulaglutide (16.7), exenatide (13.6), and PEG loxenatide (12.3).

### 3.4 Economic evaluation of GLP-1 RAs

All four GLP-1 RA drugs with the same generic name were rated with a score of 3. Exenatide had the lowest calculated daily cost (10.2 yuan) and was rated with a score of 10. Table 6 summarizes the economic scores.

**TABLE 6:** GLP-1 RA economic score results.

	Semaglutide	Dulaglutide	Exenatide microspheres	PEG loxenatide
Specification	1.5 mL:2.01 mg	1.5 mg:0.5 mL	12 mg:1.5 mL	0.5 mL:0.2 mg
Unit cost/yuan	421.34	123.35	496.25	139
Dose range	0.25–1 mg	0.75–1.5 mg	2 mg	0.2 mg
Defined daily dose (DDD)	0.11 mg	0.16 mg	—	—
Calculation daily cost calculation of DDD/yuan	23.1	13.2	11.8	19.9
Drugs with the same name	3	3	3	3
Drugs with the same indications	3.6	6.3	7	4.2
Total score	6.6	9.3	10	7.2

The economic scores are as follows: semaglutide (6.6), dulaglutide (9.3), exenatide microspheres (10), and PEG loxenatide (7.2).

### 3.5 Policy, originator, and market considerations

This category evaluates policy attributes (e.g., inclusion in Chinese health insurance and essential drug lists), originator attributes (e.g., reference drug or consistency evaluation), and market attributes (e.g., global usage and manufacturer status). These factors reflect the accessibility of drugs post-marketing. Table 7 presents the scores.

**TABLE 7:** GLP-1 RA other attribute score results.

	Semaglutide	Dulaglutide	Exenatide microspheres	PEG loxenatide
Chinese health insurance status	1.5	1.5	1.5	1.5
Chinese essential medicine status	0	0	0	0
Chinese centralized medicine status	0	0	0	0
Original/reference/consistency evaluation	1	1	1	1
Manufacturer status	1	1	1	1
Global use	1	1	1	0
Total score	4.5	4.5	4.5	3.5

The scores are as follows: semaglutide (4.5), dulaglutide (4.5), exenatide microspheres (4.5), and PEG loxenatide (3.5).

### 3.6 GLP-1 RA evaluation scores and recommendations

The evaluation results for GLP-1 RAs are presented in Table 8 and 9, with semaglutide receiving the highest score of 77.8 points, followed by dulaglutide with 76.3 points, exenatide microspheres with 70.2 points, and PEG loxenatide with 62.9 points. Descriptive statistics across the two rating rounds demonstrated that semaglutide and dulaglutide exhibited minimal variation between rounds (semaglutide: 77.55 ± 0.21 to 77.75 ± 0.21; dulaglutide: 75.4 ± 0.71 to 75.6 ± 1.30), indicating high consistency and agreement between raters. Exenatide microspheres and PEG loxenatide showed larger adjustments (exenatide microspheres: 72.3 ± 2.69 to 68.5 ± 5.23; PEG loxenatide: 64.6 ± 3.11 to 66.55 ± 5.66), reflecting refinements following expert consensus discussions, particularly in the economic and policy-related dimensions. These statistics illustrate the stability and convergence of the scoring process through structured multidisciplinary evaluation. The inter-rater reliability analysis demonstrated excellent consistency between the two raters in both rounds. Cronbach’s Alpha was 0.946 in the first round and improved to 0.980 in the second round, indicating a high degree of agreement. The results reflect the robustness of the structured consensus process and the stability of the scoring framework across rounds.

**TABLE 8:** GLP-1 RA total score results.

	Semaglutide	Dulaglutide	Exenatide microspheres	PEG loxenatide	*F*	*P*
Pharmaceutical properties	23	19.5	20.5	19.5	Null	Null
Effectiveness	27	26.3	21.6	20.4		
Safety	16.7	16.7	13.6	12.3		
Economy	6.6	9.3	10	7.2		
Other considerations	4.5	4.5	4.5	3.5		
Total score	77.8	76.3	70.2	62.9		

**TABLE 9:** The results of t-test between the two groups were compared.

	Semaglutide&Dulaglutide	Semaglutide& Exenatide microspheres	Semaglutide& PEG loxenatide	Dulaglutide and Exenatide microspheres	Dulaglutide& PEG loxenatide	Exenatide microspheres and PEG loxenatide
*t*	0.601	1.593	2.217	1.649	2.239	2.404
*P*	0.574	0.172	0.077	0.160	0.075	0.061

Based on the *Rapid Guide to Drug Evaluation and Selection for Medical Institutions in China* (*Second Edition*), semaglutide, dulaglutide, and exenatide microspheres are “strongly recommended”, while PEG loxenatide is “weakly recommended” or “not recommended”, depending on the availability of alternative drugs. The thresholds for recommendations were based on expert consensus and were designed to provide clear decision-making criteria, required a total score above 70 for a strong recommendation, thereby ensuring that only drugs meeting rigorous clinical and economic criteria were prioritized for inclusion.

## 4 Discussion

This study is the first to systematically and quantitatively assess the clinical value of once-weekly GLP-1 RAs marketed in China, utilizing a methodology grounded in the relevant Guideline. By implementing a rigorously designed quantitative record form, we address the existing gap in comprehensive, structured evaluations of these agents in clinical practice. Our systematic approach provides robust evidence to support informed, transparent, and reproducible drug selection decisions by medical institutions. The resulting ranking—semaglutide > dulaglutide > exenatide microspheres > PEG loxenatide—aligns with established efficacy and safety profiles (Xie et al., 2023), and our refined scoring method ensures objectivity and practical relevance for both policymakers and clinicians.

The results indicated minimal differences in the safety profiles of the four drugs, with the primary distinctions observed in hypoglycemic efficacy and cardiovascular benefits (Teng et al., 2024; Petrova et al., 2024). Dulaglutide and semaglutide, which offer cardiovascular benefits (Yanlan et al., 2022), demonstrated significant pharmacological and efficacy advantages. Conversely, exenatide microspheres excelled in terms of economic value. Based on these findings, dulaglutide and semaglutide were categorized as “strongly recommended”, whereas exenatide microspheres and PEG loxenatide were classified as “weakly recommended.” Medical institutions may retain or phase out the latter two drugs based on specific clinical needs.

This study introduced several methodological innovations. First, in the effectiveness evaluation, the clinical efficacy of each drug was quantitatively assessed using clinical trial data while incorporating the guideline-recommended level for each drug. Semaglutide, which exhibited the highest glucose-lowering efficacy (Hu et al., 2023), was assigned the top score of 10. The scores for other drugs were calculated using the formula: Drug score = (Glucose-lowering efficacy of semaglutide)/(Glucose-lowering efficacy of the evaluated drug) × 10. This approach enabled precise scoring of effectiveness based on glucose-lowering efficacy. Second, for the economic evaluation, the study accounted for variations in glucose-lowering efficacy and patient tolerance across products, which influence individual maintenance dosages. Economic evaluation was conducted using the daily dosage assessment of the Defined Daily Dose (DDD) value (Bindel and Seifert, 2025), thereby minimizing the impact of individual differences on the economic assessment of the drugs.

The use of quantitative scoring in comprehensive drug evaluation provides a multi-dimensional assessment framework, enabling accurate scoring of selected drugs. However, the results of such evaluations are not static and may evolve in response to changing decision-making needs. Factors influencing evaluation outcomes include the variations of different drug specifications, updates to guideline consensus and evidence-based data, the emergence of new adverse reactions, fluctuations in drug prices, adjustments to national health insurance policies and the essential medicines list, implementation of national drug policies, and developments in manufacturing enterprises. Additionally, the weights assigned to various dimensions within the evaluation system are dynamically adjusted to reflect changes in national assessment indicators and the external environment. The Supplementary Data data on drug prices, insurance attributes, and corporate credibility were incorporated into the evaluation using a scoring system aligned with the other evaluation dimensions. For example, drug prices were assessed in relation to their affordability and availability within China’s healthcare system, and corporate credibility was scored based on publicly available data on manufacturer performance and regulatory compliance.

In comparison to international HTA models, the Chinese quantitative scoring model exhibits several distinct features. For example, the United Kingdom’s National Institute for Health and Care Excellence (NICE) emphasizes cost-utility analysis through incremental cost-effectiveness ratios (ICERs) per quality-adjusted life year (QALY), but offers limited flexibility in dynamically adjusting assessment weights (Thokala et al., 2020). Germany’s Institute for Quality and Efficiency in Healthcare (IQWiG) centers on comparative effectiveness and categorizes levels of added benefit, without formal dynamic weighting (Büsch et al., 2022). Similarly, the Canadian Agency for Drugs and Technologies in Health (CADTH) focuses on real-world evidence and budget impact analyses, but employs a comparatively static weighting system (Binder et al., 2022). Japan’s Center for Outcomes Research and Economic Evaluation for Health (C2H) incorporates cost-effectiveness assessments selectively, primarily for high-cost or innovative technologies. While Japan has introduced post-listing cost-effectiveness evaluation, integration of dynamic pricing and market factors remains at an early stage and is not as flexible as in some other systems. Like many HTA frameworks, the Japanese model does not formally include manufacturer credibility as a core evaluation criterion (Shiroiwa, 2020). By contrast, the Chinese model’s dynamic adjustment of dimension weights and explicit inclusion of corporate credibility represent a more adaptive and holistic approach. However, this flexibility may also present challenges in maintaining consistency and transparency compared to more standardized international HTA frameworks.

To maintain the relevance and accuracy of drug evaluations, medical institutions should update evaluation results regularly and dynamically. They should also work toward establishing drug selection evaluation standards and operational guidelines tailored to their specific organizational needs. Incorporating these standards into pharmacy practice will enhance objectivity and accuracy in decision-making processes related to drug selection. By adopting such practices, institutions can provide decision-makers with reliable tools to make informed choices regarding drug selection and management (Janknegt et al., 1996; Shi et al., 2020; Chaojun et al., 2022; Shuning et al., 2021).

While this framework is intentionally designed to accommodate future evidence updates and policy changes, several limitations of the present evaluation should be noted. First, this mini-HTA relied on published literature and policy documents, rather than individual-level RWD. This limitation may affect the generalizability of our findings to broader patient populations in real-world clinical practice. Secondly, potential bias introduced by a weighting process that relies on expert opinion in establishing an evaluation system. Finally, there are challenges in obtaining comprehensive cost data for economic evaluation, which in this paper only calculates the minimum daily treatment cost through DDD. Future research should incorporate individual-level RWD and further refine the weighting system to improve robustness and generalizability.

## Data Availability

The original contributions presented in the study are included in the article, further inquiries can be directed to the corresponding author.

## References

[B1] American Diabetes Association (2022). Standards of medical care in Diabetes—2022. Diabetes Care 45 (1), S8–S255. 10.2337/dc22-S008 34964872

[B2] American Diabetes Association (2024). Summary of revisions: standards of care in Diabetes—2024. Diabetes Care 47 (1), S5–S10. 10.2337/dc24-SREV 38078579 PMC10725800

[B3] Author anonymous (2021). IDF diabetes atlas. 10th edition.

[B4] Barrios M. Guilera G. Nuño L. Gómez-Benito J. (2021). Consensus in the Delphi method: what makes a decision change? Technol. Forecast Soc. Change 163, 120484. 10.1016/j.techfore.2020.120484

[B5] Bethel M. A. Patel R. A. Merrill P. Lokhnygina Y. Buse J. B. Mentz R. J. (2018). Cardiovascular outcomes with glucagon-like peptide-1 receptor agonists in patients with type 2 diabetes: a meta-analysis. lancet Diabetes and Endocrinol. 6 (2), 105–113. 10.1016/S2213-8587(17)30412-6 29221659

[B6] Bindel L. J. Seifert R. (2025). Daily defined dose-costs have a stronger influence on antibacterial drug prescriptions in Germany than bacterial resistance: economic factors are more important than scientific evidence. Schmiedeb. Arch. Pharmacol. 398 (3), 2909–2921. 10.1007/s00210-024-03435-7 39302420 PMC11920358

[B7] Binder L. Ghadban M. Sit C. Barnard K. (2022). Health technology assessment process for oncology drugs: impact of CADTH changes on public payer reimbursement recommendations. Curr. Oncol. 29 (3), 1514–1526. 10.3390/curroncol29030127 35323327 PMC8947453

[B8] Bode B. W. Brett J. Falahati A. Pratley R. E. (2011). Comparison of the efficacy and tolerability profile of liraglutide, a once-daily human GLP-1 analog, in patients with type 2 diabetes≥ 65 and< 65 years of age: a pooled analysis from phase III studies. Am. J. Geriatric Pharmacother. 9 (6), 423–433. 10.1016/j.amjopharm.2011.09.007 22055210

[B9] Boustani M. A. Pittman Iv I. Yu M. Thieu V. T. Varnado O. J. Juneja R. (2016). Similar efficacy and safety of once‐weekly dulaglutide in patients with type 2 diabetes aged≥ 65 and< 65 years. Diabetes, Obes. Metabolism 18 (8), 820–828. 10.1111/dom.12687 27161178 PMC5089646

[B10] Büsch C. A. Krisam J. Kieser M. (2022). A comprehensive comparison of additional benefit assessment methods applied by institute for quality and efficiency in health care and european society for medical oncology for time-to-event endpoints after significant phase III trials—A simulation study. Value Health 25 (11), 1853–1862. 10.1016/j.jval.2022.05.015 35778324

[B11] Chaojun X. Bingnan R. Caihui G. Yue Z. Zhanjun D. (2022). Multicriteria quantitative evaluation practice of oral proton pump inhibitors. Chin. J. Mod. Appl. Pharm. 39 (2), 242–248.

[B12] Chinese Diabetes S. Chinese Diabetes Society (2020). Consensus recommendations on utilizing glucagon-like peptide-1 (GLP-1) receptor agonists in the treatment of type 2 diabetes mellitus. Zhonghua Nei Ke Za Zhi 59 (11), 836–846. 10.3760/cma.j.cn112138-20200704-00646 33120487

[B13] Gerstein H. C. Colhoun H. M. Dagenais G. R. Diaz R. Lakshmanan M. Pais P. (2019). Dulaglutide and cardiovascular outcomes in type 2 diabetes (REWIND): a double-blind, randomised placebo-controlled trial. Lancet 394 (10193), 121–130. 10.1016/S0140-6736(19)31149-3 31189511

[B14] Hu S. Su X. Fan G. (2023). Efficacy and tolerability of the subcutaneous semaglutide for type 2 diabetes patients: an updated systematic review and meta-analysis. Diabetol. Metab. Syndr. 15 (1), 218. 10.1186/s13098-023-01195-7 37891683 PMC10612199

[B15] Janknegt R. van der Kuy A. Declerck G. Idzikowski C. (1996). Hypnotics: drug selection by means of the system of objectified judgement analysis (SOJA) method. Pharmacoeconomics 10, 152–163. 10.2165/00019053-199610020-00007 10163418

[B16] Kapitza C. Nosek L. Jensen L. Hartvig H. Jensen C. B. Flint A. (2015). Semaglutide, a once‐weekly human GLP‐1 analog, does not reduce the bioavailability of the combined oral contraceptive, ethinylestradiol/levonorgestrel. J. Clin. Pharmacol. 55 (5), 497–504. 10.1002/jcph.443 25475122 PMC4418331

[B17] Lau J. Bloch P. Schäffer L. Pettersson I. Spetzler J. Kofoed J. (2015). Discovery of the once-weekly glucagon-like peptide-1 (GLP-1) analogue semaglutide. J. Med. Chem. 58 (18), 7370–7380. 10.1021/acs.jmedchem.5b00726 26308095

[B18] Linong J. Dajin Z. Tianpei H. Lulu C. Qiuhe J. Lixin G. (2018). Expert guidance on clinical applications of GLP-1 recept or agonists. Chin. J. Of Diabetes 26 (05), 353–361.

[B19] Lund A. Knop F. K. Vilsbøll T. (2014). Glucagon-like peptide-1 receptor agonists for the treatment of type 2 diabetes: differences and similarities. Eur. J. Intern Med. 25 (5), 407–414. 10.1016/j.ejim.2014.03.005 24694879

[B20] Manandhar B. Ahn J.-M. (2015). Glucagon-like Peptide-1 (GLP-1) analogs: recent advances, new possibilities, and therapeutic implications. J. Med. Chem. 58 (3), 1020–1037. 10.1021/jm500810s 25349901 PMC4329993

[B21] Mannucci E. Dicembrini I. Nreu B. Monami M. (2020). Glucagon-like peptide-1 receptor agonists and cardiovascular outcomes in patients with and without prior cardiovascular events: an updated meta-analysis and subgroup analysis of randomized controlled trials. Diabetes Obes. Metab. 22 (2), 203–211. 10.1111/dom.13888 31595657

[B22] Marsico F. Paolillo S. Gargiulo P. Bruzzese D. Dell'Aversana S. Esposito I. (2020). Effects of glucagon-like peptide-1 receptor agonists on major cardiovascular events in patients with type 2 diabetes mellitus with or without established cardiovascular disease: a meta-analysis of randomized controlled trials. Eur. Heart J. 41 (35), 3346–3358. 10.1093/eurheartj/ehaa082 32077924

[B23] Marso S. P. Daniels G. H. Brown-Frandsen K. Kristensen P. Mann J. F. Nauck M. A. (2016a). Liraglutide and cardiovascular outcomes in type 2 diabetes. N. Engl. J. Med. 375 (4), 311–322. 10.1056/nejmoa1603827 27295427 PMC4985288

[B24] Marso S. P. Bain S. C. Consoli A. Eliaschewitz F. G. Jódar E. Leiter L. A. (2016b). Semaglutide and cardiovascular outcomes in patients with type 2 diabetes. N. Engl. J. Med. 375 (19), 1834–1844. 10.1056/NEJMoa1607141 27633186

[B25] Martelli N. Hansen P. van den Brink H. Boudard A. Cordonnier A. L. Devaux C. (2016). Combining multi-criteria decision analysis and mini-health technology assessment: a funding decision-support tool for medical devices in a university hospital setting. J. Biomed. Inf. 59, 201–208. 10.1016/j.jbi.2015.12.002 26705065

[B26] Petrova L. Andreevska K. Parvova I. Petkova V. (2024). Systematic review of the efficacy and safety of GLP-1 receptor agonists in the treatment of patients with type 2 diabetes mellitus. Pharmacia 71, 1–17. 10.3897/pharmacia.71.e132148

[B27] Ping W. Jing Z. (2022). A case of acute attack of cholecystitis induced by polyethylene glycol loxenatide. Chin. J. Hosp. Pharm. 42 (21), 2311–2313.

[B28] Pradhan R. Montastruc F. Rousseau V. Patorno E. Azoulay L. (2020). Exendin-based glucagon-like peptide-1 receptor agonists and anaphylactic reactions: a pharmacovigilance analysis. Lancet Diabetes and Endocrinol. 8 (1), 13–14. 10.1016/S2213-8587(19)30382-1 31806579

[B29] Sha L. Lina Z. Jiao X. Youjia L. (2022). Literature analysis of adverse drug reactions induced by dulaglutide. Chin. J. Hosp. Pharm. 42 (09), 930–934.

[B30] Shi X. Zhao R. Li F. Liu C. Gao J. Zhao K. (2020). Reflections on the establishment of clinical comprehensive evaluation mechanism for drugs in China. China Pharm., 2828–2833.

[B31] Shiroiwa T. (2020). Cost-effectiveness evaluation for pricing medicines and devices: a new value-based price adjustment system in Japan. Int. J. Technol. Assess. Health Care 36 (3), 270–276. 10.1017/S0266462320000264 32419677

[B32] Shuning Z. Chunli H. Jianzhong Z. (2021). Benefit-risk assessment in drugs evaluation (in Chinese). Chin. J. Clin. Pharmacol. 37 (13), 1757–1763.

[B33] Society C. D. (2021). Guideline for the prevention and treatment of type 2 diabetes mellitus in China (2020 edition). Chin J. Diabetes Mellitus 13 (4), 315–409.

[B34] Teng Y. Fan X. Yu R. Yang X. (2024). Evaluation and comparison of efficacy and safety of tirzepatide, liraglutide and SGLT2i in patients with type 2 diabetes mellitus: a network meta-analysis. BMC Endocr. Disord. 24 (1), 278. 10.1186/s12902-024-01805-z 39719583 PMC11668020

[B35] Thokala P. Carlson J. J. Drummond M. (2020). HTA’d in the USA: a comparison of ICER in the United States with NICE in England and Wales. J. Manag. Care and Specialty Pharm. 26 (9), 1162–1170. 10.18553/jmcp.2020.26.9.1162 32857653 PMC10391099

[B36] Wong-Rieger D. Tsai I. C. Tan J. Y. Wu D. B.-C. Yu D. Keetley A. (2025). Redefining value assessment and healthcare funding priorities for medicines: the journey to patient-centric decision making in APAC – a systematic literature review. Int. J. Technol. Assess. Health Care 41 (1), e28. 10.1017/S0266462325000224 40365699 PMC12086590

[B37] Xie Z. Hu J. Gu H. Li M. Chen J. (2023). Comparison of the efficacy and safety of 10 glucagon-like peptide-1 receptor agonists as add-on to metformin in patients with type 2 diabetes: a systematic review. Front. Endocrinol. (Lausanne) 14, 1244432. 10.3389/fendo.2023.1244432 37701904 PMC10493284

[B38] Yanlan L. A. I. Aiwen H. Guanxu C. Tingting C. Lijun Z. Xiaolan L. (2022). Cardiovascular benefits of SGLT-2 inhibitors and GLP-1 receptor agonists in type 2 diabetes: a systematic review and network meta-analysis. J. Pharm. Pract. Serv. 40 (4), 354–358.

[B39] Yihong S. Kang C. Xin C. Weijun G. Yuanlin G. Yijun L. (2021). Expert consensus on the management of diabetic patients with cardiovascular diseases (in Chinese). Chin. J. Intern Med. 60 (5), 421–437. 10.3760/cma.j.cn112138-20201208-00999 33906272

[B40] Zhao Z. G. Dong Z. J. Liu J. P. (2023). A quick guideline for drug evaluation and selection in Chinese medical institutions (the second edition). Her. Med. 42 (4), 447–456.

[B41] Zhao Z. G. Dong Z. J. Liu J. P. (2020). A quick guideline for drug evaluation and selection in Chinese medicalinstitutions. Her. Pharm. 39, 1457–1465.

